# Austria-based real-world data on bevacizumab in newly diagnosed epithelial ovarian cancer

**DOI:** 10.1007/s00508-022-02005-2

**Published:** 2022-02-10

**Authors:** Irina Tsibulak, Stephan Polterauer, Alexander Reinthaller, Christian Schauer, Jürg Berger, Christian Marth

**Affiliations:** 1 Department of Obstetrics and Gynecology, Medical University Innsbruck, Anichstraße 35, 6020 Innsbruck, Austria; 2 Department of Obstetrics and Gynecology, Comprehensive Cancer Center Vienna, Medical University Vienna, Währinger Gürtel 18–20, 1090 Vienna, Austria; 3 Department of Gynecology, Konventhospital Barmherzige Brueder, Marschallgasse 12, 8020 Graz, Austria; 4 Roche Austria GmbH, Engelhorngasse 3, 1210 Vienna, Austria

**Keywords:** Ovarian carcinoma, Survival, Adverse events, Anti-angiogenesis

## Abstract

**Background:**

Front-line maintenance therapy with bevacizumab demonstrates high efficacy and safety in epithelial ovarian cancer, as already shown in large phase III trials; however, the corresponding study populations are often not fully representative of patients in clinical routine. In this Austria-based multicenter study, we aimed to explore the real-world outcomes of bevacizumab use in front-line treatment of ovarian cancer, including patients with comorbidities and poor performance status.

**Patients:**

This study is an open label single arm multicenter noninterventional trial and included patients with newly diagnosed advanced epithelial ovarian cancer, who were treated with platinum-based chemotherapy and were candidates for receiving bevacizumab according to the product label. Data collection started in the third quarter of 2012 and ended in the third quarter of 2018.

**Results:**

In this study 50 patients were included and 575 adverse events were reported for 90% of the patients. The majority of the adverse events were mild (47%) or moderate (37%). The most common adverse events were hypertension (60%), anemia (48%), leukopenia (42%), thrombocytopenia (36%), neutropenia (36%) and proteinuria (26%). A relation to bevacizumab was documented only for 10.3% of all adverse events. In almost 50% of all adverse events, no intervention was needed and bevacizumab treatment had to be interrupted only in 3.3% of all adverse events. The median progression-free survival was 1.3 years (95% CI 1.1–1.8).

**Conclusion:**

The routine use of front-line bevacizumab for advanced ovarian cancer is associated with high efficacy comparable with that obtained in randomized phase III clinical trials; however, hypertension and proteinuria were reported significantly more often in our Austria-based real-world population.

**Supplementary Information:**

The online version of this article (10.1007/s00508-022-02005-2) contains supplementary material, which is available to authorized users.

## Introduction

Epithelial ovarian cancer (EOC) is the seventh most common cancer among women in Austria and belongs to the leading causes of cancer death in females in the western world [1]. In 2018, 761 women were diagnosed with EOC in Austria and 520 of them succumbed to this malignant disease [1]. EOC is most likely to be diagnosed at advanced stages, requiring extensive surgical cytoreduction as well as platinum-based systemic treatment [2]. During the last two decades, targeted therapies and the concept of maintenance therapy became increasingly more important in the management of EOC. Angiogenesis plays a crucial role in ovarian cancer progression and therefore represents an essential therapeutic target in treatment of EOC. During the last years, several anti-angiogenic agents have been investigated in EOC and of these only bevacizumab, a monoclonal antibody targeting vascular endothelial growth factor, has been introduced in the treatment of ovarian cancer. Based on the results of ICON‑7 and GOG0218 trials, front-line maintenance therapy with the angiogenesis inhibitor bevacizumab was approved in Europe and since 2018 it has also been approved in the USA [3, 4]. The most common adverse events of this agent are hypertension and proteinuria; however, these rarely lead to bevacizumab discontinuation [3, 4]. At the present time, front line maintenance therapy with bevacizumab is broadly used and demonstrates high efficacy and safety, as already shown in large phase III trials [3–5].

It is without any doubt that these trials provide excellent evidence of efficacy; however, the corresponding study populations are often not fully representative of patients in clinical routine, as prospective clinical trials tend to have strict eligibility criteria. In this Austria-based noninterventional multicenter study, we aimed to explore the real-world outcomes of bevacizumab use in front-line treatment of EOC, including patients with comorbidities and poor performance status.

## Patients, material and methods

The present study was designed as an open label single arm multicenter non-interventional trial (sponsored by Hoffmann-La Roche under ML28355; ClinicalTrials.gov number NCT01788995). The participating centers included gynecological departments at the Medical University of Innsbruck, Medical University of Vienna, and Konventhospital Barmherzige Brüder in Graz. The ethics committee of the Medical University of Innsbruck, Austria approved the present study (IRB approval#: UN4727_NIS; 21.06.2012).

Patients aged ≥ 18 years with newly diagnosed advanced EOC, who were treated with platinum-based chemotherapy and were candidates for receiving bevacizumab according to the product label, were eligible for the study. Patients received bevacizumab 15 mg/kg body weight every 3 weeks combined with standard front-line therapy with carboplatin and paclitaxel and then continued as single agent maintenance therapy. The inclusion of patients was based on the physician’s decision. There were no specific exclusion criteria as long as patients received bevacizumab according to the drug approval.

In this trial 60 patients were planned to be included. Written informed consent was obtained for every patient. Clinical pathological characteristics were collected using an electronic Case Report Form (eCRF). Data collection started in the third quarter of 2012 and ended in the third quarter of 2018. Descriptive statistics including the occurrence in total and in percent, the minimum and maximum values, the median and the arithmetic means, were performed. No hypothesis was predefined regarding the primary and secondary objectives of the study. Progression-free survival was defined as the interval between first dose of front-line therapy until documented disease progression. Missing data were replaced with “NA” (not applicable).

## Results

Due to sponsor’s decision, the recruitment was stopped as 57 patients with newly diagnosed advanced EOC were recruited in this study. Of the patients 7 were excluded from the analysis as they did not receive a first-line platinum-based chemotherapy in combination with bevacizumab, so that 50 patients who received systemic treatment with carboplatin/paclitaxel and bevacizumab were included in the analysis. Bevacizumab starting dose was 15 mg/kg body weight every 3 weeks for all patients. The median number of bevacizumab cycles was 15 cycles (range 1–30 cycles). Treatment interruptions caused by adverse effects or planned surgical procedures were reported for 33 (66%) patients. The median treatment duration was 1.0 years (range 0.1–1.7 years) and the median follow up time was 2.25 years (range 0.42–4.92 years).

The median age of the patients was 60 years, while almost 80% were 50 years and older. Most of the patients (78%) presented with Eastern Cooperative Oncology Group (ECOG) performance status 0 and 22% with ECOG status 1 (Supplementary Table 1).

Of the patients 64% presented with Fédération Internationale de Gynécologie et d’Obstétrique (FIGO) stage IIIc disease, 18% with FIGO stage IIIb and 14% had a stage IV disease. The detailed histology was not routinely documented in each center; however, the cohort of 29 patients at the Department of Obstetrics and Gynecology of the Medical University of Innsbruck consisted of 1 patient with endometrioid ovarian carcinoma, 1 patient with low grade serous ovarian cancer and 27 patients with high grade serous carcinoma.

More than 55% (*n* = 28) of patients had no residual tumor after cytoreductive surgery, while in 30% (*n* = 15) the complete resection was not achieved and in 14% (*n* = 7) residual disease was not documented.

For 24% (*n* = 12) of patients pre-existing hypertension was documented. The summary of pre-existing comorbidities is presented in Supplementary Table 2 and 20 patients (40%) had no pre-existing comorbidities.

Overall, 575 adverse events (AEs) were reported for 45 (90%) of the 50 patients. The majority of the reported AEs were mild (*n* = 268, 47%) or moderate (*n* = 215, 37%) and 92 severe AEs were documented (16%). The most common AEs were hypertension occurring in 60% of patients (*n* = 30), anemia (*n* = 24, 48%), leukopenia (*n* = 21, 42%), thrombocytopenia (*n* = 18, 36%), neutropenia (*n* = 18, 36%) and proteinuria (*n* = 13, 26%) (Table 1); however, a relation to bevacizumab was documented only for 10.3% of all AEs (*n* = 59), while 33.4% (*n* = 192) were related to carboplatin and 38.3% (*n* = 220) were related to paclitaxel.

**Table 1:** Most common adverse events per patient

Adverse Event	*n* Patients	% Patients
Hypertension	30	60.0
Anemia	24	48.0
Leukopenia	21	42.0
Neutropenia	18	36.0
Thrombocytopenia	18	36.0
Proteinuria	13	26.0
Urinary tract infection	13	26.0
Drug hypersensitivity	11	22.0
Nausea	9	18.0
Infection	6	12.0
Diarrhea	5	10.0
Fatigue	5	10.0
Vomiting	5	10.0
Constipation	4	8.0
Pain in extremity	4	8.0
General physical health deterioration	4	8.0
Thrombocytosis	4	8.0
Urinary tract infection bacterial	4	8.0

In almost 50% of all AEs, no intervention was needed, 35.5% of AEs were treated with medication therapy and in 3.3% (*n* = 23) of all AEs bevacizumab therapy had to be interrupted. Of these 3.3%, only 1% (*n* = 7) of AEs were associated with bevacizumab (proteinuria in *n* = 3 and hypertension in *n* = 4).

**Fig. 1:**
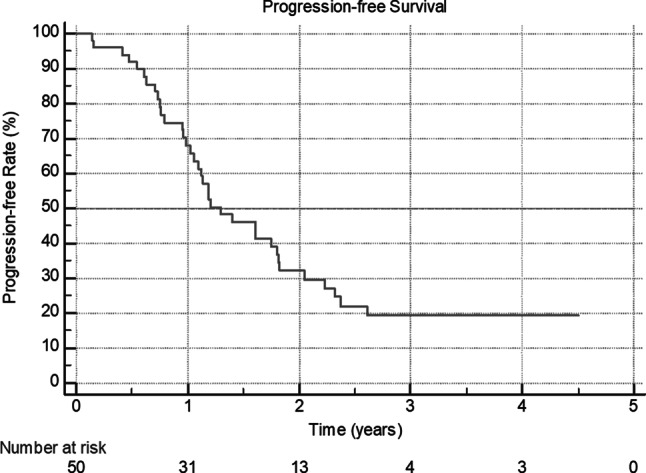
Kaplan-Meier curve for progression-free survival

Progressive disease was documented for 36 (72%) patients, while median progression-free survival (PFS) was 1.3 years (95% CI 1.1–1.8) (Fig. 1). Death occurred in 11 (22%) patients, no median overall survival (OS) could be calculated (not reached).

## Discussion

In this multicenter noninterventional study of bevacizumab in newly diagnosed ovarian cancer we evaluated the routine clinical use in three Austrian centers. The combination of bevacizumab and carboplatin/paclitaxel was well tolerated, as already shown in the large phase III trials (GOG0218, ICON7) [3, 4]. In accordance with these trials, most adverse events were typical of the chemotherapy regimen (such as anemia, neutropenia, thrombocytopenia) and were not related to bevacizumab; however, in our population, hypertension and proteinuria were reported significantly more often: hypertension in 60% in our study vs. 26% in ICON7 and 23% in GOG0218; proteinuria in 26% vs. 5% and 1.6%, respectively [3, 4]. Similar results were showed for hypertension in the single arm ROSiA trial (55% of patients), but still much lower incidence of proteinuria (7.6% of patients) was documented in ROSiA as compared to our study [5]. This high incidence of hypertension as an adverse event could be due to the fact that 24% of our patients already had a pre-existing hypertension. We assume that the high incidence of proteinuria is not due to comorbidities, as no pre-existing proteinuria was reported before bevacizumab in our cohort; however, proteinuria was reported for 20% of patients in OSCAR study, which was a UK-based observational study of front-line bevacizumab in advanced EOC [6]. In our study, no gastrointestinal perforation and no wound healing complications occurred, probably due to the small population size, as these complications had a low incidence rate in larger trials: gastrointestinal perforation in > 1% of patients in ICON7, 2.6% in GOG0128 and 2% in the observational OSCAR study; wound healing complication in 5%, 3% and 2% of patients, respectively [3, 4, 6]. Approximately 80% of AEs in our cohort were resolved or showed improvement and bevacizumab had to be interrupted in only 3.3%, so that no new safety signals emerged.

The median time to progression in our population was 15.6 months, which is comparable to the GOG218, ICON7 and OSCAR study results (14.1, 16.0 and 15.4 months, respectively) [3, 4, 6]. On the other hand, the PFS of 15.6 months in our study is significantly shorter than PFS of 25.5 months in the international ROSiA study [5]; however, in ROSiA bevacizumab treatment was continued up to 24 months and 23% of patients had a stage I–IIIA disease, whereas the median number of bevacizumab cycles in our study was 15 cycles and 96% of our patients presented with FIGO stage IIIb–IV disease.

The main limitations of our study are the small patient size and the lack of a control arm.

To our knowledge, this is the first study which reflects real-world Austria-based clinical practice, including patients with pre-existing morbidities. Our results show encouraging evidence that the routine use of front-line bevacizumab for advanced ovarian cancer is associated with high safety and efficacy comparable with those obtained in randomized phase III clinical trials; however, hypertension and proteinuria were reported significantly more often in our Austria-based real-world population.

## Supplementary Information

Supplementary Table 1: Eastern Cooperative Oncology Group (ECOG) performance status. Supplementary Table 2: Pre-existing comorbidities.
