# Supplementary material for: Austria-based real-world data on bevacizumab in newly diagnosed epithelial ovarian cancer
Source: Wien Klin Wochenschr. 2022 Feb 10;134(19-20):693–6. doi: 10.1007/s00508-022-02005-2 (PMC9606045; doi:10.1007/s00508-022-02005-2)
Supplement: Supplementary file 1 — Supplementary Table 1: Eastern Cooperative Oncology Group (ECOG) performance status. Supplementary Table 2: Pre-existing comorbidities. [file 508_2022_2005_MOESM1_ESM.docx]

Supplementary material

Supplementary Table 1: ECOG performance status

| **ECOG** | **n patients** | **% patients** |
| --- | --- | --- |
| 0 | 39 | 78.0% |
| 1 | 11 | 22.0% |
| Total | 50 | 100.0% |

Supplementary Table 2: Pre-existing comorbidities

| **Pre-existing comorbidities** | **n patients** | **% patients** |
| --- | --- | --- |
| Hypertension | 12 | 24.0% |
| Hypothyroidism | 11 | 22.0% |
| Cholelithiasis | 6 | 12.0% |
| Depression | 6 | 12.0% |
| Drug hypersensitivity | 6 | 12.0% |
| Hypercholesterolemia | 6 | 12.0% |
| Hiatus hernia | 5 | 10.0% |
| Atrial fibrillation | 4 | 8.0% |
| Constipation | 4 | 8.0% |
| Deep vein thrombosis | 3 | 6.0% |
| Gastritis | 3 | 6.0% |
| Goiter | 3 | 6.0% |
| Hydronephrosis | 3 | 6.0% |
| Obesity | 3 | 6.0% |
| Renal cyst | 3 | 6.0% |
